# Recurrence of Differentiated Thyroid Carcinoma During Full TSH Suppression: Is the Tumor Now Thyroid Hormone Dependent?

**DOI:** 10.1007/s12672-014-0204-z

**Published:** 2014-10-08

**Authors:** Paul J. Davis, Aleck Hercbergs, Mary K. Luidens, Hung-Yun Lin

**Affiliations:** 1 Department of Medicine, Albany Medical College, Albany, NY USA; 2 Pharmaceutical Research Institute, Albany College of Pharmacy and Health Sciences, One Discovery Drive, Rensselaer, NY 12144 USA; 3 Department of Radiation Oncology, Cleveland Clinic, Cleveland, OH USA; 4 PhD Program for Cancer Biology and Drug Discovery, College of Medical Science and Technology, Taipei Medical University, Taipei, Taiwan

## Abstract

Well-standardized primary treatment and long-term management of differentiated thyroid carcinoma (DTC) include lowering or suppression of host thyrotropin (TSH) with exogenous L-thyroxine (T_4_). This treatment recognizes the trophic action of TSH on DTC cells. Suppression of endogenous TSH with T_4_ is continued in recurrent disease. However, T_4_ can induce proliferation of follicular and papillary thyroid carcinoma cell lines and of other human carcinoma cells. The proliferative mechanism is initiated at a cell surface receptor for T_4_ on integrin αvβ3, a receptor by which the hormone also inhibits p53-dependent apoptosis in tumor cells. In recurrent DTC with satisfactory suppression of endogenous TSH, we discuss here the possibility that the tumor is no longer TSH dependent and that T_4_ has become a critical growth factor for the cancer.

## Management of Differentiated Thyroid Carcinoma

Differentiated thyroid carcinoma (DTC) accounts for about 90 % of thyroid cancers [40]. Initial and long-term management guidelines provided by the American Thyroid Association have worked to standardize the surgical, radioablative, and medical treatment of differentiated thyroid carcinoma (DTC) [16] and have drawn a number of substantial comments [63, 70, 73]. A key feature of DTC treatment is the lowering in low risk or the suppression in higher risk DTC patients of circulating endogenous pituitary thyrotropin (TSH). Reduction of patient TSH release is achieved with exogenous thyroid hormone (L-thyroxine, T_4_). TSH is a growth factor for papillary and follicular thyroid cancer, and successful TSH suppression therapy assumes a functional TSH receptor (TSHR) in thyroid cells whose activity is minimized by the dearth of circulating TSH. The risks and benefits of TSH suppression therapy have been reviewed in detail by Biondi and Cooper [4].

Recurrence of disease occurs in 15 % or more of DTC patients [52], with a higher incidence of recurrence in patients over the age of 65 years [34]. Recurrence may be a product of patient noncompliance with the TSH suppression regimen, but, in the compliant patient, the molecular basis of recurrent tumor is not clear. The tumor cell TSH receptor (TSHR) may be a hyperfunctional mutant in DTC (2726) and thus may respond over time in residual cancer cells to low-but-detectable circulating levels of TSH. However, the frequency of TSHR mutations in DTC is variable [9]. The state of p53 in the remaining tumor cells may also determine lesion aggressiveness [57]. That a silenced TSHR is often present in the case of *undifferentiated* thyroid cancers [28, 54] is certainly consistent with the existence of endogenous growth or regulatory factors that are alternatives to TSH and may support thyroid tumor cell proliferation. There are a number of examples of such factors, including the epidermal growth factor (EGF) axis [39, 82] and platelet-derived growth factor (PDGF) [29, 83]. While it is important to growth of various cancers, the EGF receptor in papillary DTC in terms of abundance and affinity appears to be the same in tumoral and adjacent normal thyroid tissue [53]. On the other hand, PDGF may promote lymphatic metastases in DTC [83], and vascular endothelial growth factor (VEGF) supports development of lymph node metastases from DTC, at least in certain ethnic populations [10]. Insulin-like growth factor 1 (IGF-1) is a trophic factor for DTC [69]. Thyroid transcription factor 1 (TTF-1) may be localized to the nucleus in differentiated thyroid carcinoma with increased frequency in the settings of recurrent or persistent disease [27]. Hormonal regulation of *TTF*-*1* has not been well studied [5].

Certain gene mutations occur in DTC but have had variable success as predictors of recurrence. BRAF mutations, particularly V600E, have drawn particular attention in DTCs [1, 18, 26, 37, 66].

Given the above information, the possibility should be considered that additional endogenous growth factors exist for DTC in the absence of TSH that will support recurrence. One such factor is thyroid hormone (see next section) which has been shown to have proliferative effects on follicular and papillary thyroid carcinoma cells [48].

## Proliferative and Anti-apoptotic Actions of Thyroid Hormone at Integrin αvβ3 on DTC and Other Tumor Cells

Integrin αvβ3 is a structural protein of the plasma membrane that binds specific extracellular matrix proteins and is critical to cell-cell and cell-matrix interactions [64, 76]. It is amply expressed by and activated in cancer cells and rapidly dividing blood vessel cells [21]. The protein has been recently appreciated to contain small molecular receptor sites for androgen [45], resveratrol [43], and thyroid hormone [2, 21, 41]. Acting at its receptor on αvβ3, thyroid hormone (L-thyroxine, T_4_; 3,5,3′-triiodo-L-thyronine, T_3_) has been shown to be pro-angiogenic [12, 20, 51] and to induce proliferation in a variety of human cancer cells. The latter includes glioblastoma [46], lung [55], pancreas [81], and kidney [79] cells. The thyroid hormone receptor on the integrin has no structural homologies with the classical nuclear thyroid hormone receptor (TR) [14, 41]. The αvβ3-based actions of the hormone are nongenomic, in that they are independent of the T_3_-TR interactions that are the basis of the genomic mechanism of hormone action. From the integrin, however, T_4_ can modulate genomic actions of T_3_ [22] by affecting the trafficking of TR from cytoplasm to nucleus [8], the state of phosphorylation of TR [23], and the formation of TR-coactivator protein complexes [44]. The presence of αvβ3 has been confirmed in the many solid tumor cell lines with which we have worked (HY Lin; unpublished observations), regardless of putatively aggressive phenotype. Studies by others in nonthyroid solid tumor cell lines have indicated that αvβ3 confers recurrence behavior when other factors, such as chemokine CCL2 [49] or matrix metalloproteinase-9 (MMP-9) [33], are also present.

As noted above, we have shown that follicular thyroid cancer cells and papillary thyroid carcinoma cells [48] proliferate in vitro in response to T_4_ at physiologic free concentrations. The proliferative response requires MAPK (ERK1/2) activation and is blocked by RGD peptide, indicating the effect is initiated at the hormone receptor site on αvβ3. These observations raise the possibility that endogenous thyroid hormone can be a growth factor for undiagnosed thyroid cancer. That in patients with an established diagnosis of DTC there may be recurrent local and metastatic thyroid cancer despite successful suppression of endogenous TSH release with pharmacologic thyroid hormone may have trophic effects on the recurrent tumor. The proliferative effect of T_4_ (10^−10^ M free hormone) on thyroid cancer cells has been observed in nonmutant cell lines, including TTF-1-expressing cells, in Ret/PC1-positive cells [48], and in heterozygous BRAF(V600E)/wt cells (HY Lin: unpublished observations).

A second approach which has been used to examine the proliferative actions of thyroid hormone on DTC cells in vitro involves tetraiodothyroacetic acid (tetrac), the naturally occurring deaminated analog of T_4_. Tetrac inhibits the trophic effects of T_4_ and T_3_ on a variety of tumor cells [46, 47, 55], including papillary and follicular cancer cells [48]. This action depends upon inhibition by tetrac of the binding of thyroid hormone to integrin αvβ3 [20]. Tetrac formulations—including nanoparticulate tetrac that is excluded from the cell interior—have been shown to arrest the growth of human follicular thyroid carcinoma xenografts [78], xenografts of medullary thyroid carcinoma [80], and other human solid tumor xenografts [58, 79, 81]. Such observations are consistent with actions of thyroid hormone as a trophic factor for cancers, including DTC. But, it should be noted that tetrac has anti-cancer and anti-angiogenic effects that are additive to its activity as an inhibitor of iodothyronine binding to the integrin [21].

Thyroid hormone has a number of other actions that may support solid tumor growth. The hormone is pro-angiogenic [19, 51] by several mechanisms, including enhancement of the angiogenic activities of VEGF, FGF2 (bFGF), and PDGF [51]. Interestingly, the hormone may inhibit proliferative action of IGF-1 in myoblasts [36] but amplifies IGF-1 signaling in chondrocytes [74] and other cells [61]. This suggests that thyroid hormone effects on this growth factor are complex and may be tissue specific. The hormone stimulates miR-21 production that enhances metastasis [35], as does hormonal action to repress miR-17 [50]. The hormone causes transcription of a panel of metalloproteinase genes (*MMP*-*2*, *MMP*-*3* [60], and *MMP*-*9* [15, 60, 65]) that support tumor dispersal/metastasis. A number of these actions are initiated at integrin αvβ3. Differential expression of genes in cancer cells that code for regulatory proteins important to the cell cycle is also stimulated by thyroid hormone [71]. Thyroid hormone has anti-apoptotic actions in a variety of cells [11, 13, 25, 42, 47, 48, 62, 67, 72, 84]. We have shown that the hormone can block pharmacologic induction of p53-dependent apoptosis [48] by interfering with Ser-15 phosphorylation of p53. We would point out that the hormone has also been reported to cause apoptosis, but the models typically involve hyperthyroidism in intact animals or very high doses of the hormone in vitro, e.g., 250–500 μM [75, 77]. Finally, the pro-angiogenic activity of thyroid hormone [51] may be interpreted as a support mechanism for cancers.

The affinity of the thyroid hormone receptor on plasma membrane integrin αvβ3 is higher for T_4_ than T_3_ [2]. Functionally, this means that supraphysiologic amounts of T_3_ are required to induce proliferation in vitro, as shown, for example, in human glioblastoma cells [46]. In contrast, concentrations of free T_4_ of 10^−10^ M or less are proliferative. This has raised the possibility of lowering tumor patient circulating T_4_ levels via suppression of patient TSH with antithyroid drug therapy [32] or exogenous T_3_ [30, 31]. This strategy is discussed in the next section.

## Is Recurrent DTC in Part an Expression of Actions of Thyroid Hormone?

This question is not a denial of the role of TSH in support of differentiated thyroid cancer or of the utility of the lowering of TSH or its suppression in this clinical setting. We would agree that in the large majority of DTC patients, the relative importance of TSH and the proliferative and/or anti-apoptotic activity of thyroid hormone used to suppress endogenous TSH strongly favors the contribution of endogenous TSH and is a requirement for its suppression.

On the other hand, the mechanisms for recurrence or relapse of well-differentiated papillary or follicular thyroid cancer in the fully TSH-suppressed patient have not been satisfactorily defined. That thyroid hormone, notably as T_4_, stimulates tumor cell proliferation and is anti-apoptotic has been repeatedly demonstrated [42, 47, 48] in a variety of cell lines in vitro, including follicular and papillary thyroid cancer cells, as noted above. The αvβ3 integrin that mediates such hormonal activity is amply expressed by cancer cells and by endothelial cells involved in tumor-related angiogenesis. This mechanism is independent of nuclear TR.

There are several strategies that may be discussed to test the concept that T_4_ used to suppress endogenous TSH may contribute adversely to tumor behavior in patients with recurrent DTC. First, TSH-suppressive administration of T_3_ may be substituted for L-thyroxine. T_3_ may be administered sufficiently frequently to assure suppression of endogenous TSH and achieve frank reduction in circulatingT_4_ levels. The clinical endpoint is number and size of recurrent tumors. This approach anecdotally has resulted in decreased size of certain cancers [30, 31], but has not been tested against DTC.

Second, when recurrent DTC occurs in the setting of full TSH suppression with T_4_, the treatment with T_4_ may be interrupted for several months. The TSH may be allowed to rise into the normal range or higher, and symptomatic hypothyroidism would be managed with sufficient L-thyroxine to abolish symptoms. The intent of reinstitution of T_4_ treatment is not to return serum TSH to the normal range, but only to relieve symptoms. The clinical endpoint again is number and size of recurrent tumors. Without satisfactory documentation that, in fact, T_4_ has replaced TSH as a growth factor for the tumor, this approach cannot be used.

Third, thyroid hormone responsiveness of biopsied recurrent, invasive DTC might be tested in one or model systems. One such approach involves xenografting of tumor biopsy cells into the chick chorioallantoic membrane (CAM) system, with biomarker response testing of the cells to TSH and, separately, to T_4_. The results may then direct subsequent management. This is a personalized medicine approach that we have elsewhere suggested may be used in the future to define pharmacodynamics of multiple chemotherapeutic agents against recurrent cancers of various types [24]. Tumor cell radiosensitivity can also be evaluated in the CAM.

A final point for discussion is that deaminated thyroid hormone analogs are agents that suppress pituitary TSH secretion. Tetrac [7] and triac [6, 7, 38] have been shown to be effective inhibitors of TSH secretion and have been considered for use in the setting of thyroid cancer. As discussed above, tetrac also blocks the proliferative actions of T_4_ on tumor cells that are initiated at αvβ3 [21, 41] and triac also has been shown to inhibit nongenomic actions of T_4_ [19]. The use of either of these agents may suppress host TSH and, independently of this action, inhibit DTC tumor cell proliferation. The approach has an important disadvantage. Unmodified tetrac and triac are taken up by nonmalignant cells and are thyromimetic in such cells [56], albeit low grade. Such thyromimetic effects in noncancer cells may have interrupted the proposed commercial development of unmodified tetrac as a TSH-suppressing drug. In contrast, nanoparticulate tetrac—a compound in which the tetrac is covalently bound to a biodegradable nanoparticle [3, 21]—is not internalized by any cells and expresses its anti-tumor and anti-angiogenic activity exclusively at αvβ3 in the plasma membrane. Limited to the cell exterior, however, nanoparticulate tetrac is not a suppressor of pituitary TSH secretion.

A section of the guidelines of the American Thyroid Association for management of DTC includes an essay on directions for future research [16]. Emphases are placed on disruption of oncogenic signaling pathways, particularly MAPK, on modulation of proliferation and apoptosis and on development or use of inhibitors of angiogenesis. These themes appear to us to be very relevant to the possibility we raise here that TSH-suppressive doses of T_4_ in the patient population with recurrent, aggressive DTC may then contribute to the clinical growth of the tumor. The proliferative actions of T_4_ initiated at αvβ3 are MAPK dependent [46]; T_4_ is anti-apoptotic [42, 47, 48] and T_4_ is pro-angiogenic [20, 51]. Thus, consideration of the elimination of these actions of T_4_ in cancer cells that express αvβ3 has some attractiveness. Studies to determine whether T_4_ is a proliferative factor in xenografts have not yet been carried out, but induced or spontaneous hypothyroidism in patients with glioblastoma [32], renal cell carcinoma [68], nonthyroidal head and neck cancers [59], and breast carcinoma [17] has favorably affected tumor behavior.
